# Molecular Dynamic Simulation Reveals Structure Differences in *APOL1* Variants and Implication in Pathogenesis of Chronic Kidney Disease

**DOI:** 10.3390/genes13081460

**Published:** 2022-08-16

**Authors:** Richard Mayanja, Christopher Kintu, Oudou Diabate, Opeyemi Soremekun, Olugbenga Oluseun Oluwagbemi, Mamadou Wele, Robert Kalyesubula, Daudi Jjingo, Tinashe Chikowore, Segun Fatumo

**Affiliations:** 1 The African Computational Genomics (TACG) Research Group, Medical Research Council/Uganda Virus Research Institute and London School of Hygiene & Tropical Medicine Uganda Research Unit, Entebbe 31405, Uganda; 2 Department of Immunology and Molecular Biology, College of Health Sciences, Makerere University, Kampala 10101, Uganda; 3 African Center of Excellence in Bioinformatics (ACE-B), University of Science, Technique and Technologies of Bamako (USTTB), Bamako 3206, Mali; 4 Molecular Bio-Computation and Drug Design Laboratory, School of Health Sciences, University of KwaZulu-Natal, Durban 4041, South Africa; 5 Department of Computer Science and Information Technology, Sol Plaatje University, Kimberley 8301, South Africa; 6 Department of Internal Medicine and Department of Physiology, Makerere University, Kampala 10101, Uganda; 7 African Center of Excellence in Bioinformatics (ACE-B), Makerere University, Kampala 10101, Uganda; 8 MRC/Wits Developmental Pathways for Health Research Unit, Department of Paediatrics, Faculty of Health Sciences, University of the Witwatersrand, Johannesburg 2050, South Africa; 9 Sydney Brenner Institute for Molecular Bioscience, Faculty of Health Sciences, University of the Witwatersrand, Johannesburg 2193, South Africa; 10 Department of Non-Communicable Disease Epidemiology (NCDE), London School of Hygiene and Tropical Medicine, London WC1E 7HT, UK

**Keywords:** molecular dynamic simulation, molecular docking, chronic kidney disease, *APOL1*, mutation

## Abstract

Background: According to observational studies, two polymorphisms in the apolipoprotein L1 (*APOL1*) gene have been linked to an increased risk of chronic kidney disease (CKD) in Africans. One polymorphism involves the substitution of two amino-acid residues (S342G and I384M; known as G1), while the other involves the deletion of two amino-acid residues in a row (N388 and Y389; termed G2). Despite the strong link between *APOL1* polymorphisms and kidney disease, the molecular mechanisms via which these *APOL1* mutations influence the onset and progression of CKD remain unknown. Methods: To predict the active site and allosteric site on the *APOL1* protein, we used the Computed Atlas of Surface Topography of Proteins (CASTp) and the Protein Allosteric Sites Server (PASSer). Using an extended molecular dynamics simulation, we investigated the characteristic structural perturbations in the 3D structures of *APOL1* variants. Results: According to CASTp’s active site characterization, the topmost predicted site had a surface area of 964.892 Å^2^ and a pocket volume of 900.792 Å^3^. For the top three allosteric pockets, the allostery probability was 52.44%, 46.30%, and 38.50%, respectively. The systems reached equilibrium in about 125 ns. From 0–100 ns, there was also significant structural instability. When compared to G1 and G2, the wildtype protein (G0) had overall high stability throughout the simulation. The root-mean-square fluctuation (RMSF) of wildtype and variant protein backbone Cα fluctuations revealed that the Cα of the variants had a large structural fluctuation when compared to the wildtype. Conclusion: Using a combination of different computational techniques, we identified binding sites within the *APOL1* protein that could be an attractive site for potential inhibitors of *APOL1*. Furthermore, the G1 and G2 mutations reduced the structural stability of *APOL1*.

## 1. Introduction

Kidney disease affects between 11% and 15% of the world’s population [1]. Every year, millions of people die prematurely as a result of kidney-related diseases. Damaged kidneys are unable to filter blood adequately, process toxic wastes, and manage the excess fluid buildup in the human body [2,3]. Chronic kidney disease (CKD), defined as a gradual loss in kidney function for more than 3 months, has been found to be most common in people of African descent, accounting for approximately 15% of the population [4,5].

The apolipoprotein L1 (*APOL1*) gene has been linked to a fourfold increase in the risk of developing CKD in African-Americans [6,7].

**Figure 1:**
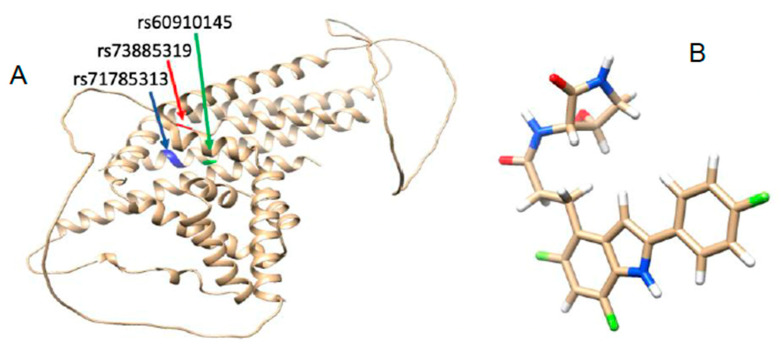
The 3D structure of *APOL1* protein highlighting the point of mutation (**A**) and 3D structure of potential *APOL1* inhibitor (**B**). For the G1 mutations, SNPs rs60910145 and rs73885319 correspond to I384M and S342G respectively, while, for G2, SNP rs1785313 corresponds to N388del: Y389del.

*APOL1* is involved in the innate immunity of trypanosomes and other lysing infecting flagellated parasites [8,9]. Parasite-induced resistance to *APOL1* activities, on the other hand, has resulted in the emergence of two nonsynonymous coding variants (G1(S342G:I384M) and G2(N388del:Y389del) [10]. Carrying the two risk *APOL1* alleles increases the risk of CKD development, progression, and severity by 70% [11]. This has also contributed to rapid disease development and end-stage renal disease (ESRD) in people of recent African descent [11]. As a result, in addition to protecting against trypanosomes, *APOL1* (see Figure 1A) plays a negative role [12]. The two most common treatments for kidney failure are dialysis and kidney transplantation [13]. Unfortunately, these are expensive and unavailable in resource-constrained environments [14]. Sabins (2020) established a number of novel chemicals for the treatment of kidney diseases, most notably focal segmental glomerulosclerosis (FSGS) and/or nondiabetic kidney disease (NDKD) [15]. We investigated the mechanistic impact of a ligand on the dynamics of the *APOL1* protein using a newly synthesized compound (Compound **1**) (see Figure 1B). As a result, this study was designed to gain insight into the impact of mutations on the 3D architecture of the *APOL1* protein and to posit a possible mechanism of action of a potential *APOL1* inhibitor, in order to gain further insight into the etiology of CKD.

## 2. Methodology

### 2.1. Protein and Ligand Preparation

The AlphaFold database was used to obtain the human *APOL1* protein structure accession number O14791 [16]. Molegro molecular viewer was used to refine the structures [17]. The *APOL1* G1 variants, rs73885319 and rs60910145, were created by replacing Ser at position 342 with Gly (Ser342Gly) and Ile at position 384 with Met (Ile384Met) in CHIMERA using the “Swapaa” command line [18]. The *APOL1* G2 (rs71785313) variant is the result of a 6 bp deletion; thus, the mutation was introduced in the structure by removing Asn at position 388 and Tyr at position 389 and using the join command [19]. The steps taken to prepare the ligand were as follows: the 2D structure was drawn with Marvin sketch [20], after which energy minimization was carried out on the ligand using the energy optimization tool within Avogadro software [21]. We used the GAFF forcefield and the steepest descent algorithm to obtain the most stable form and lowest energy of the ligand. The energy-minimized structure was then used for molecular docking in Chimera [22].

### 2.2. Molecular Dynamic Simulation

The simulation was carried out using the AMBER force field, FF18SB [23]. We used the General Amber Force Field (GAFF) and the restrained electrostatic potential (REP) to describe the atomic charges of APIND. Amber’s Leap module was used to perform system neutralization and hydrogen atom addition. An orthorhombic box of TIP3P water molecules surrounding all of the *APOL1* atoms at a distance of 9 Å [24] was also used for system solvation. We performed system minimization in 2000 steps using a restriction potential of 500 kcal/mol. This was followed by 1000 steps of full minimization with no constraints. In all, we undertook two minimization process. The first minimization, partial minimization, was undertaken to relieve bad van der Waals contact in the surrounding solvent while keeping the solute, i.e., the protein, restrained. The second minimization process, full minimization, was undertaken to relieve bad contacts in the whole system. The system was gradually heated from 10 to 273 K at 50 ps using a Langevin thermostat at a collision frequency of 1.0 ps^−1^. After equilibration, we heated each system for 50 ps while maintaining a constant temperature of 300 K and pressure of 1 bar (isobaric-isothermal ensemble, NPT using Berendsen barostat) with a timestep of 2 fs. We used the SHAKE algorithm [25] within the AMBER software to constrain (NTC = 2) all bonds involving hydrogen. This step is important as it removes the highest-frequency oscillation in the system and that of hydrogen vibrations. The PTRAJ module of Amber 14 was used for additional analyses such as root-mean-square deviation (RMSD), root-mean-square fluctuation (RMSF), and radius of gyration [26], as in previous publications [27]. We created data plots with the ORIGIN analytical tool. AMBER’s molecular mechanics/Poisson–Boltzmann surface area (MM/GBSA) module was used to estimate thermodynamic calculations, as described below.

G_bind_ = G_complex_ − (G_receptor_ + G_inhibitor_), (1)

ΔG_bind_ = ΔG_gas_ + ΔG_sol_ − TΔS, (2)

ΔG_gas_ = ΔE_int_ + ΔE_ele_ + ΔE_vdW_, (3)

ΔG_sol_ = ΔG_ele,sol_(GB) − ΔG_np,sol_, (4)

ΔG_np,sol_ = γSASA + β (5)

where ΔG_gas_ represents the total gas-phase energy calculated by intermolecular energy (ΔE_int_), electrostatic energy (ΔE_elel_), and van der Waals energy (ΔE_vdW_), ΔG_sol_ represents the solvation energy, TΔS represents the entropy change, ΔG_ele,sol(GB)_ describes the polar desolvation energy, ΔG_np,sol_ describes the nonpolar desolvation energy, γ is the surface tension proportionality constant and is set to 0.0072 kcal·mol^−1^·Å^−2^, β is a constant equal to 0, and SASA is the solvent-accessible surface area (Å^2^).

## 3. Results

### 3.1. APOL1 Structural Elucidation

The 3D structure of *APOL1* was obtained from the AlphaFold protein structure database [16], and the active sites were predicted using the Computed Atlas of Surface Topography of Proteins (CASTp) server [28]. The CASTp server identifies topographic features, measures area and volume, and computes imprint using the α shape method developed in computational geometry [29]. According to CASTp’s active site characterization, the top predicted site had a surface area of 964.892 Å^2^ and a spatial volume of 900.792 Å^3^. We used PASSer (Protein Allosteric Sites Server) to evaluate potential APOL1 allosteric pockets. Allostery probabilities for the top three allosteric pockets were 52.44%, 46.30%, and 38.50%. Other descriptors of these pockets are reported in Table 1.

### 3.2. Mutation-Structural Perturbation of APOL1

**Figure 2:**
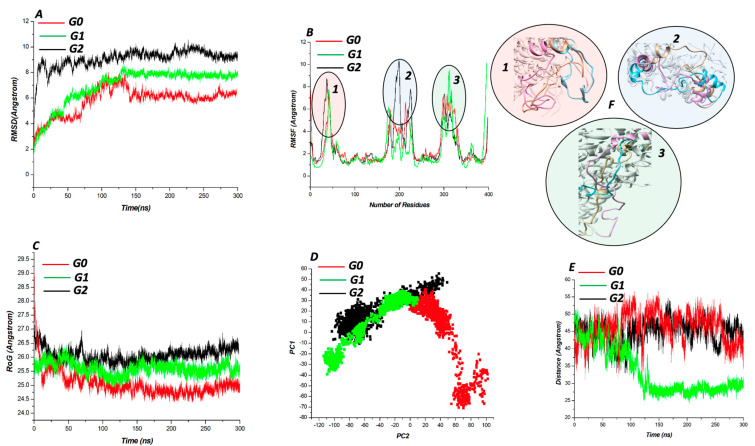
Backbone RMSDs depicted as a function of time for G0 (red), G1 (green), and G2 (black) (**A**). Cα fluctuation of G0 (red), G1 (green), and G2 (black) (**B**). RoG plot of G0 (red), G1 (green), and G2 (black) (**C**). PCA scatter plots depicting a distinct separation of motions between G0 (red), G1 (green), and G2 (black) (**D**). Intermolecular distance plot of G0 (red), G1 (green), and G2 (black) (**E**). The 3D structures of highly fluctuating region in the system (**F**).

Molecular docking was carried out using AutoDock vina tools in-built in Chimera. A grid box with coordinate (center: *x* = 12.622, *y* = 45.876, *z* = 24.735; size: *x* = 21, *y* = 29, *z* = 30) was set around the binding sites of *APOL1* wildtype and mutant proteins predicted by CASTp to accommodate the ligand (APIND). The binding affinity of the wildtype and mutant proteins upon binding to APIND was as follows: G0, −7.9 kcal/mol; G1, −8.4 kcal/mol; G2, −9.1 kcal/mol. Because mutations within a protein can potentially alter its 3D structure, we investigated the molecular behavior of the Cα of the wild, G1, and G2 variants using an extended molecular dynamics simulation. The metrics RMSD, RoG, RMSF, and PCA were used to evaluate the MD trajectories [29]. RMSD analysis provides information about the protein’s conformational change and overall stability during the MD simulation. While all of the systems reached equilibration around 125 ns, there was significant structural instability from 0–100 ns (Figure 2A). The G0 protein had an overall high stability throughout the simulation, with an average RMSD value of 5.79 Å, as expected, while the G1 and G2 proteins had average RMSD values of 6.96 Å and 9.09 Å, respectively. The RMSF is a measure of individual residue displacement during the simulation. Backbone Cα fluctuations expressed as the RMSF of wildtype and variant proteins revealed that the Cα of the variants had a high structural fluctuation when compared to the wildtype. The G0, G1, and G2 average Cα fluctuations were 2.27 Å, 2.63 Å, and 2.76 Å, respectively (Figure 2B). Importantly, high fluctuation was observed in regions other than the mutation point, leading us to believe that the mutation has a “distant effect” on the structure of *APOL1* (Figure 2F). The radius of gyration, which is a measure of the RMS average of the distance of the atoms from the protein’s center, supported the RMSD analysis, as the wildtype protein had less atomic gyration than the G1 and G2 variants (Figure 2C).

PCA is a parameter used in unraveling a protein’s conformational changes and mobility during simulation. The PCA highlighted the disparate motions of the wildtype and variant proteins along two major components, as observed in the RMSD and RMSF analyses (PC1 and PC2). The variants showed greater dispersion along these principal components (G0: −48.15, 9.75; G1: −47.81, 18.21; G2: 40.57, 2.68) (Figure 2D). We used ORIGIN to extrapolate these coordinates by identifying the minimum and maximum data points in each principal components of the systems. Furthermore, the system’s intermolecular distance supported the structural instability caused by the variants (Figure 2E). This intermolecular distance was calculated for the whole system by using the center of mass.

#### APIND Alters the Structure of *Apol1*

**Figure 3:**
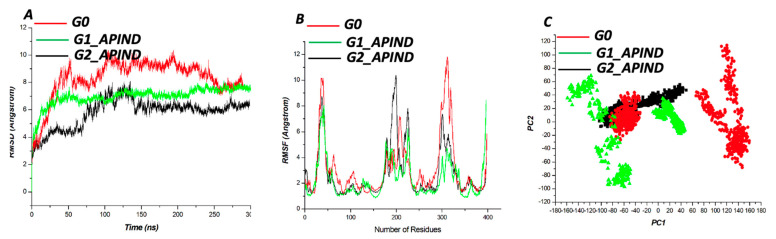
Backbone RMSDs are depicted as a function of time for the wildtype protein (G0) and bound variants (G1 and G2) (**A**). Cα fluctuation for the wildtype protein (G0) and bound variants (G1 and G2) (**B**). PCA scatter plots depicting a distinct separation of motions for the wildtype protein (G0) and bound variants (G1 and G2) (**C**).

Distinctive from Figure 2, where we explored the structural changes in the variants without ligand binding, we undertook a protein–ligand simulation to investigate the potential of APIND to mitigate the effect of the structural distortion in *APOL1* caused by mutation (Figure 3). Our findings show that APIND binding reduced the structural average RMSD of G1 and G2 variants, lowering the structural average RMSD from 6.96 Å to 6.83 Å for G1 and from 9.09 Å to 5.780 Å for G2 variants (Figure 3A). The Cα fluctuation followed a similar trend. However, some regions demonstrated high flexibility, presumably due to the presence of loops (Figure 3B). The RMSD trend was observed in the PCA analysis of G1 and G2 (Figure 3C).

**Figure 4:**
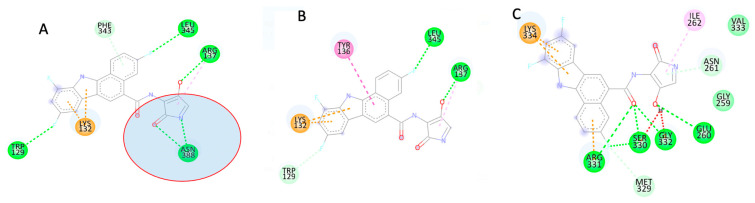
Two-dimensional structures of G0_APIND (**A**), G1_APIND (**B**), and G2_APIND (**C**) interactions.

We elucidated the mechanistic interaction of APIND within the predicted active site of *APOL1* protein using the rs71785313 variant as a model to posit a possible mechanism of action of the potential *APOL1* inhibitor (APIND). We discovered that, in the wildtype protein (G0), APIND forms a strong hydrogen bond with ASN388 (red circle; Figure 4A); however, when ASN388 is deleted in G2, this interaction is lost. This is likely one of the mechanisms via which APIND stabilizes the 3D structure of *APOL1* and inhibits the protein. 

The MM/GBSA technique for calculating free binding energy could provide insight into protein–ligand interaction systems. As a result, we looked into the time-dependent bond interactions between critical residues in the active sites of *APOL1* variants and APIND. The total binding energies of G1 and G2 upon APIND binding were −10.65 kcal/mol and −20.876 kcal/mol, respectively, according to thermodynamic calculations (Table 2).

Figure 4 depicts how this energy contributed to the thermodynamic process. 

## 4. Discussion

*APOL1* variants have been linked to an increased risk of developing CKD, particularly in African-Americans [30]. Understanding how a protein works and the functional effects of its modification, such as through site-directed mutations, can be aided by studying its structure [31]. Knowledge of protein structure also allows us to understand molecules that bind to proteins, decipher disease pathophysiology, and identify potential treatment targets [27]. A single amino-acid change in a protein’s structure can have disastrous functional consequences [32]. As in previous studies [33], using a combination of computational techniques such as computational modeling, molecular docking, and molecular dynamics (MD) simulations, we identified binding sites within the *APOL1* protein that could be an attractive site for potential *APOL1* inhibitors [34]. These approaches aided in our understanding of the structure of APOL1-G0, as well as the effect of G1 and G2 variations on protein structure and dynamics. As a result, we investigated the distinctive structural perturbations in the 3D structures of *APOL1* variants. The introduction of the G1 and G2 mutations reduced the structural stability of *APOL1*. The findings of this study are supported by other experimental studies that have alluded to structural instability in G1 and G2 variants of *APOL1*, affecting *APOL1* interactions with other proteins within the cell [35]. Protein residues are implicated in various activities, including protein structure and function [36]. Mutations can cause decreased structure stability, resulting in misfolding and a loss of protein function [37]. Changes in protein conformation and stability provide information about the deviation of backbone atoms from their starting structure during MD simulation. The *APOL1* protein–ligand interaction improved structural stability, according to our findings. This could be because protein interactions with small ligands frequently result in an increase in protein thermostability due to the coupling of binding and unfolding equilibrium [38]. Although kidney diseases are ostensibly a global health issue, renal replacement is prohibitively expensive and difficult to obtain [38]. In the quest for more treatment options, we discovered that variation-mediated protein conformational changes may impair *APOL1* variants’ ability to regulate or interact with other downstream proteins such as VAMP8 and SNARE. As a result, this may lead to the development and progression of CKD. This is a computer-based study in which we looked at only one compound as a potential drug. As a result, in silico, in vivo, and in vitro studies are needed to screen various compounds for their potential as drug candidates for kidney-related diseases [33].

## Figures and Tables

**Table 1:** Allosteric site descriptors of *APOL1* allosteric sites.

	Score	Druggability	Volume	Hydrophobicity	Residues
Pocket 1	0.023	0.002	300.261	43.667	N154,L147,L21,K132,L85,C13,V349,S342,Q82,D395,F265,V254,Q134,H130,V338,L258
Pocket 2	−0.034	0.002	126.023	44.875	N154,V349,Q239,H360,H241,Q237,Y354,R157,V350,K357,K233,L161,V244,S356,L243,A240,L158,L371,T236,V353,L352
Pocket 3	−0.037	0.001	3.333	13.167	V350,A5,Y351,E90,C13,G270,L6,L12,T272,R8,E348,F265,Y354,L266,A269,V9,L347,F344, E92,L86

**Table 2:** Thermodynamic calculations of G1 and G2 variants upon APIND binding.

Energy Component	G1_APIND	G2_APIND
∆E_vdW_ (kcal/mol)	−10.23	−19.084
∆E_ele_ (kcal/mol)	20.78	−40.092
∆G_GB_ (kcal/mol)	4.65	65.1435
ESURF (kcal/mol)	−3.89	−5.086
∆G_gas_ (kcal/mol)	6.44	−99.98
∆G_sol_ (kcal/mol)	4.41	60.846
∆G_bind_ (kcal/mol)	−10.65	−20.876

## Data Availability

The data presented in this study are available in the manuscript.
